# Effect of Unmeasured Time Hours on Occupational Noise Exposure Assessment in the Shipbuilding Process in Korea

**DOI:** 10.3390/ijerph18168847

**Published:** 2021-08-22

**Authors:** Jaewoo Shin, Seokwon Lee, Kyoungho Lee, Hyunwook Kim

**Affiliations:** 1 Department of Public Health, Graduate School, The Catholic University of Korea, Seoul 06591, Korea; sjw1063@cmcnu.or.kr (J.S.); swlee23@korea.kr (S.L.); 2 Center for Occupational and Environmental Medicine, Seoul Saint Mary’s Hospital, The Catholic University of Korea, Seoul 06591, Korea; 3 Environmental Health Research Department, National Institute of Environmental Research, Incheon 22689, Korea; 4 Division of Population Health Research, Department of Precision Medicine, National Institute of Health, KDCA, Cheongju 28160, Korea; 5 Department of Preventive Medicine, College of Medicine, The Catholic University of Korea, Seoul 06591, Korea

**Keywords:** break period, hearing protection, noise, occupational noise exposure, shipbuilding process, unmeasured time hours

## Abstract

Occupational noise is known to be one of the most hazardous risk factors, frequently exceeding the exposure limit thus causing hearing loss and other health outcomes among many field workers in various industries and workplaces. This study aims to characterize the levels of occupational noise exposure during the daily working hours and break periods (sampling preparation and lunch break), identify work-related characteristics affecting the noise exposure levels when including or excluding the break periods and finally determine the most effective approach for occupational noise exposure assessment by using the Korean and U.S. OSHA’s guidelines. A total of 1575 workers employed by a large shipbuilding company participated in this study, and the historical exposure datasets of noise dosimeters, collected from 2016 to 2018, were classified by characteristics. A threshold level (TL) for the noise dosimeter was set as a value of 80 dBA during the break periods, including the preparation time for sampling instruments and one hour for the lunch break. The shipbuilding workers were exposed to high levels of occupational noise during the break periods, especially for those working in heating, grinding, and power processes in the painting-related departments. Out of 1575 samples, most cases were related to the preparation time (*N* = 1432, 90.9%) and lunch break (*N* = 1359, 86.9%). During the break time, the levels of noise exposure were measured depending on task-specific characteristics. When including the break time, the noise levels increased by approximately 1 dBA during the break, combining 0.8 dBA in the lunch hours and 0.2 dBA for the preparation of the sampling instrument. When excluding the break time, the levels of noise exposure collected using a Korean Occupational Safety and Health Administration (KOSHA) guide tended to be underestimated compared to those using the U.S. OSHA method. When including the break times, the proportion of noise exposure levels exceeding the compliance exposure limit declined from 37.9% to 34.5%, indicating that the break times might affect the decrease in the noise exposure levels. Taken together, shipbuilding workers could possibly be exposed to much greater amounts of noise exposure during break times in the shipbuilding processes, and the noise exposure levels in the department of painting were high. Therefore, it is recommended that industrial hygienists collect exposure monitoring data of occupational noise one hour after their job tasks begin and then consecutively monitor the noise exposure levels for at least 6 h including the break periods for each day.

## 1. Introduction

Occupational noise exposure is one of the most important risk factors causing hearing loss among workers in a variety of industries and workplaces, and approximately 16% of manufacturing workers suffer from hearing loss with serious consequences [1], including irritation, sleep disorders, daytime sleepiness, metabolic syndrome, hypertension, and cardiovascular disease, due to acute or chronic exposure to occupational noise [2,3,4,5,6,7]. In Korea, occupational noise is known to be one of the most hazardous workplace risk factors, frequently exceeding the occupational exposure limit (OEL) during quantitative exposure assessments in a large number of industries and workplaces in the past [8,9,10].

To prevent occupational hearing loss, it is important that a comprehensive evaluation be performed to quantitatively characterize all levels of cumulative noise exposure during a full shift of fixed jobs, thus identifying the task-based exposure profiles for individual workers [11]. In general, personal noise exposure is determined as a daily hour time-weighted average (8 h-TWA) value using a cumulative noise meter measured within a radius of 30 cm from the ears of the worker over 6 h and then compared to the established OEL, 85 dBA, in Korea [12]. Most studies suggested that exposure monitoring samples consecutively collected during a full shift of working hours per day provides the most accurate noise exposure assessment [13,14,15]. The U.S. Occupational Safety and Health Administration (OSHA) suggested that noise exposure assessment be performed to collect full shift monitoring samples for at least 7 h per day while excluding any less than 1 h break time as unmeasured periods [16]. However, the Ministry of Employment and Labor (MoEL) defined the length of noise exposure monitoring samples to be over 6 h per day considering the real-world work environment in Korea [17].

In Western countries, some prestigious academic societies and governmental institutions, including the British Occupational Hygiene Society (BOHS) [18], the American Industrial Hygiene Association (AIHA) [19], and the OSHA [20], have established and validated the OEL of noise exposure and conduct occupational exposure assessments for compliance purposes to determine whether the measured levels of noise exposure exceed the established OEL. Several studies have also recommended that a comprehensive occupational exposure evaluation should access all types of physical and chemical agents, including noise, thus establishing task-specific exposure profiles among individual workers collected during daily working hours [21,22]. A previous study reported that the annual levels of occupational noise exposure were significantly decreasing when analyzing the U.S. OSHA’s Integrated Management Information System (IMIS) database, which includes all industries 1979–2013 in the North American Industry Classification System (NAICS), including shipbuilding and repair, and comparing the results of quantitative exposure measurements to the permissible exposure limit (PEL), 90 dBA, and action level (AL), 85 dBA [23].

In Korea, occupational exposure assessment has focused on the analysis of a small number of monitoring samples collected for only a few workers representing each occupation (job title) or department, instead of using similar exposure groups (SEGs) classified based on detailed qualitative information on the magnitude and frequency of noise exposure in the shipbuilding industry [24]. This approach has been used simply because detailed quantitative information on exposure profiles and associated determinants can be primarily collected during exposure monitoring events in real-world workplaces. Recent studies have shown the patterns and characteristics of underwater radiated noise from small ships and ducts using computational fluid dynamics (CFD) programs [25], and case studies have also suggested a new engineering approach to effectively reduce noise exposure levels in the work environment using a noise simulation [26,27]. However, no study has been previously performed to assess occupational noise exposures for a large group of workers engaged in shipbuilding processes in the shipyard industry using qualitative exposure information on work-related characteristics and evaluate the effect of unmeasured time hours for lunch break and instrument preparation when including or excluding break hours, on the average levels of noise exposure, thus determining whether or not they are exceeding the OELs established by the Korean and U.S. OSHA’s guidelines.

Therefore, this study aims to characterize the levels of occupational noise exposure during the break period (sampling preparation and lunch break hour) among a large number of manufacturing workers in the shipbuilding industry, identify several work-related characteristics affecting the noise exposure levels when including or excluding the break periods during the exposure monitoring, and statistically comparing the average levels of noise exposure for different groups of workers to determine whether or not they are exceeding the regulatory limit values using both Korean and the U.S. OSHA’s guidelines. In doing so, we ultimately focused on identifying the most applicable approach to noise exposure assessment while considering the work-related characteristics, patterns, and other factors of daily exposure measurements among individual workers in the shipbuilding industry in Korea.

## 2. Materials and Methods

### 2.1. Selection of Study Subjects

**Figure 1:**
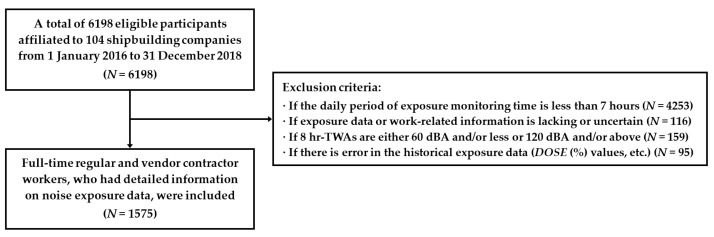
Flow diagram for selection of study subjects.

We conducted occupational exposure assessment for full-time regular and subcontracted workers affiliated with a total of 104 shipbuilding and vendor companies located in the southern area of the Republic of Korea from 1 January 2016 to 31 December 2018. We collected the exposure monitoring data using noise dosimeters for a total of 6198 workers in the targeted shipyard work environments. Of the 6198 eligible participants, their exposure data indicated an unmeasured period of less than 1 h per day (i.e., over 7 h of daily working hours), and they were included in the first step. Then, we excluded some participants in reference to Middendorf [23] if any of the following criteria were satisfied: (1) if the daily period of exposure monitoring time was less than 7 h prior to 4 p.m.; (2) if either exposure data or work-related information (e.g., department, subtask, employment type, etc.) necessary for the classification and determination of exposure profiles for individual workers was lacking or uncertain; (3) if the 8 h-TWA value was either 60 dBA and/or less or 120 dBA and/or above, and (4) if there was an error in the historical exposure data (e.g., *DOSE* (%) values, etc.). Consequently, a total of 1575 shipbuilding workers were selected as the study subjects after excluding some participants (*N* = 4623) according to the criteria mentioned above (Figure 1). This study was approved by the Institutional Review Board (IRB) of the Catholic University of Korea College of Medicine, Seoul, Republic of Korea (IRB approval No. MC13QASI0043).

### 2.2. Data Collection

We performed data cleaning and established a new database for analyzing the cumulative noise exposure data of 1575 workers using the “dBLink” package (version 3.3.0.5, Cirrus Research plc, Hunmanby, North Yorkshire, UK). The characteristics of the measuring instrument were identified and classified by the collected information on the monitoring year and dates, equipment number, etc., using the cumulative noise exposure data collected from the legal work environment measurement during 2016–2018. Historical exposure measurement data were collected using a cumulative noise dosimeter instrument (CR 110A Dozebage, Circus, EU), which was determined to be suitable for ANSIS1-25-1978. At this point, the hearing correction circuit of the noise meter was characterized by its A-type characteristics, its operation was slow, and its metric setting criteria were set to Threshold (TL) 80 dB, Exchange Rate (ER) 5 dB, Response Slow, and Criterion (C-90) according to the Ministry of Employment and Labor regulation No. 2018-62 and the U.S. OSHA’s hearing preservation program.

Furthermore, the authors collected data from their work environment measurement staff each time they performed legal work environment measurements 2016–2018 of all relevant information necessary for the classification of the noise exposure group of the participants. We also de-identified and coded the participants’ personal information (e.g., name, age, gender, date of birth, phone number, etc.). After completing data collection, cleaning, and classification, all authors carefully reviewed and cross-checked all of the collected datasets to determine if there was any miswritten error or missing or incomplete information among these study subjects. Then, the suitability was checked with an officer of the Environmental Safety Team, and the repeated processes of QA/QC were conducted to revise such misleading information in the datasets.

### 2.3. Classification of Job and Subtasks

To develop SEGs, we collated detailed information on work-related characteristics of exposure, including working environment, department, employment type, manufacturing process, job title, and subtasks, for a total of 1575 workers (full-time regular and vendor contractor) in the shipbuilding industry and interpreted the definitions and descriptions (examples) for all classified characteristics of exposure as shown in Table 1.

### 2.4. Occupational Exposure Assessment

The original timetable of occupational exposure assessment was from 8 a.m. to 5 p.m. per day, and the break periods included preparation time for exposure sampling instruments (before 8 a.m.) and 1 h for a lunch break (from 12 p.m. to 1 p.m.). We measured the levels of occupational noise exposure (dBA), including the break periods, and collated the historical exposure data measured over 7 h, including the unmeasured sampling period (less than 1 h). We assumed that the noise exposure levels measured during the full-shift sampling period were comparable to those collected during the unmeasured sampling period, and then the same value of *DOSE* (%) was applied to calculate the 8 h-TWA (i.e., *L_AVG_*) values [12].

All datasets of occupational noise exposure measurements (*N* = 1575) were classified by the factor of work-related characteristics (e.g., type of work environment, department, employment type, and job task) and then individually matched to one of the classified categories for each characteristic, depending on our professional judgment about the data input process. The values of 8 h-TWA (*L_AVG_*), *DOSE* (%), and exposure sampling periods that included the lunch break (1 h) from the individual datasets were entered into a software program, the dBLink package version 3.3 (Cirrus Research plc, Hunmanby, North Yorkshire, UK), and the same calculations were repeated using different values for 8 h-TWA, *DOSE* (%) and sampling periods between segments divided into different timetables of exposure sampling (e.g., before 8 a.m., from 8 a.m. to 4 p.m., after 4 p.m.).

Regardless of including the break periods (preparation of sampling instrument and lunch break hours), *L_AVG_* was calculated using Equations (1) and (2) with detailed exposure information on work-related characteristics (e.g., working condition, department, employment type, and job tasks) [20]. According to the KOSHA guide [12,13], the average noise level (*L_AVG_*) during the noise monitoring period was calculated from the measured data by using a value of *DOSE* (%) for lunchtime and one hour for lunchtime out of the total measurement in Equation (2). Then, each time-weighted average (TWA) value with a change in the monitoring time (increase in the unmeasured time) was also obtained using Equations (1) and (2). The abnormal time (over 8 h) was calculated by modifying the 8-hour exposure limit, 90 dBA, using Equation (3).

The first formula of cumulative equivalent noise level, *Leq* (dB), was calculated using Equation (1) as follows:

(1) $$Leq\;\left(\mathrm{dB}\right)=16.61\times \log\frac{\left(t_{1}\times 10^{\frac{SPL_{1}}{16.61}}\right)+\left(t_{2}\times 10^{\frac{SPL_{2}}{16.61}}\right)+\cdots +\left(t_{n}\times 10^{\frac{SPL_{n}}{16.61}}\right)}{t_{1}+t_{2}+\cdots +t_{n}}$$

*t*: sampling period (min), *SPL*: sound pressure level (dBA), *ER* = 5 dB.

A second formula for 8 h-TWA (*L_AVG_*) and *DOSE* (%) values (*CL* = 90, *ER* = 5 dB, *CT* = 8 h) was calculated using Equation (2) as follows:

(2) $$TWA_{T}=16.61\times \log\left(\frac{CT\times DOSE\left(\%\right)}{T\times 100\%}\right)+90\;\mathrm{dBA}\;TWA_{T}=16.61\times \log\left(\frac{DOSE\left(\%\right)}{T\times 12.5\%}\right)+90\;\mathrm{dBA}$$

$$DOSE\left(\%\right)=\left(\frac{C_{1}}{T_{1}}+\frac{C_{2}}{T_{2}}+\cdots +\frac{C_{n}}{T_{n}}\right)\times 100$$

*C*: daily working hour, *T*: permissible exposure time corresponding with measured SPL.

The third formula (*ER* = 5 dB) converted with time (*T*) and noise level (*L*) is defined as Equation (3) as follows:

(3) $$T=\frac{8}{2^{\frac{\left(L-90\right)}{5}}}$$

We compared occupational noise exposures using the U.S. OSHA method and KOSHA guideline (Table 2). When the break time was included, the average noise levels (*L_AVG_*) using the U.S. OSHA method and KOSHA guideline were the same. However, the KOSHA guide did not adjust the OEL for abnormally longer working hours (≥8 h). When excluding the break periods, the KOSHA guide tends to underestimate compared to the U.S. OSHA method because the *L_AVG_* values are evaluated by using a formula that includes *DOSE* (%) generated during the break time but excludes lunchtime (for 1 h).

### 2.5. Statistical Analysis

All statistical analyses were performed using the SPSS software package version 18.0 for Windows (IBM Corporation, Armonk, NY, USA). The level of noise exposure during the break period (preparation and lunch hours) was determined by *DOSE* (%) measured using a noise dosimeter with a threshold (TL) of 80 dBA. The value of *L_AVG_* was calculated using the measured time period (in hours) and *DOSE* (%) for each measurement classified by characteristics (work environment, department, employment type, or job task). The paired t-test was also performed to compare the *L_AVG_* values for the same measurements during different sampling timetables with or without the break periods. The paired t-test and McNemar test were applied to compare the noise exposure levels measured using the different approaches of the Korean and OSHA’s hearing conservation program (HCP) by characteristics. Finally, the intraclass correlation coefficients (ICCs) were calculated to compare the average levels of occupational noise with the OEL values of 90 dBA (8 h) and 89.2 dBA (9 h) by decreasing 1 h for daily exposure sampling timetables from the beginning to the end of exposure sampling, and the McNemar test was performed to determine the most relevant timetable among the different sampling timetables with *p*-values at a significance level of 0.05.

## 3. Results

**Figure 2:**
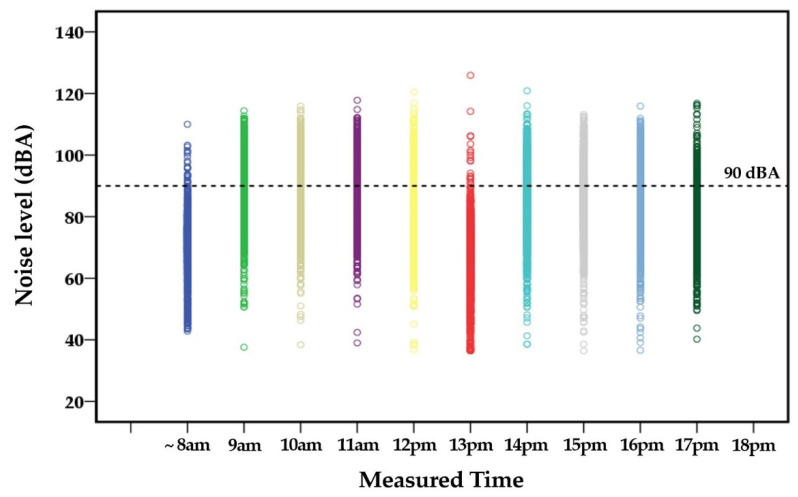
Noise exposure levels of all study subjects (*N* = 1575) at each measured time per day.

**Figure 3:**
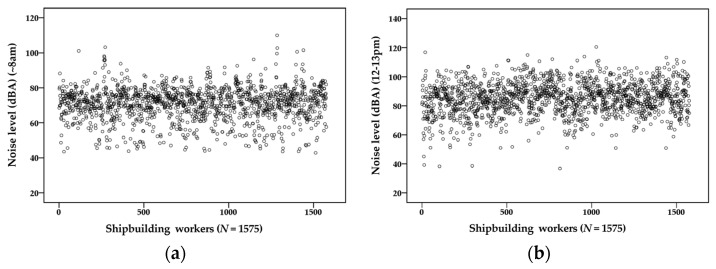
Patterns of noise exposure levels during the break time periods: (**a**) preparation of sampling instruments (~8 a.m.), (**b**) lunch break hour (12–13 p.m.).

During the break periods, including preparation time for sampling instruments (e.g., pre- and post-calibration, etc.) and the 1 h lunch break, the noise exposure levels were individually measured for a total of 1575 shipbuilding workers engaged in various job tasks. Out of 1575 exposure datasets, high levels of noise exposure were associated with preparation (*N* = 1432, 90.9%) and the lunch break (*N* = 1359, 86.9%). Figure 2 shows noise exposure levels and patterns of all study subjects (*N* = 1575) measured during the entire daily working hours, including break time periods, and the ranges of occupational noise levels for 12~13 p.m. (lunch break) were comparable to those measured for 9~11 a.m. (after beginning the daily job tasks) and 13~15 p.m. (after lunch break). Figure 3 also shows that the patterns of noise exposure measured during the break periods, (a) prior to the beginning of job tasks for the preparation of sampling instruments (~8 a.m.) and (b) lunch break hour (12~13 p.m.), frequently exceeded the OEL, 90 dBA.

Table 3 shows the average levels of noise exposure (*L_AVG_*) during the break periods by each characteristic, and they were significantly higher inside (81.8 dBA) than outside the workplaces (71.8 dBA) during the preparation time. However, *L_AVG_* was higher outside (83.5 dBA) than inside the workplaces (77.5 dBA) during the lunch breaks. The *L_AVG_* was the highest in the department of block painting (88.9 dBA) during the preparation time but was 95.9 dBA in the department of hull painting during the lunch hour and was similarly high in the block painting department (87.6 dBA) during lunch break. The levels of *L_AVG_*, 79.1 dBA, and 83.1 dBA for contractor workers collected during the preparation and lunch break hours were also significantly higher than those of the full-time regular workers, i.e., 73.9 dBA and 70.2 dBA, respectively. Among the 13 different job tasks, the highest levels of *L_AVG_* were 84.9 dBA for blasting during the preparation and 100.9 dBA for power tools during the lunch break.

In Table 4, the mean levels of occupational noise exposure by their characteristics, including and excluding the break period (preparation of instruments and lunch break hour), are described. The overall mean level of occupational noise exposure (*N* = 1575) was 88.3 dBA when excluding the break period, and it was significantly higher than 87.3 dBA when including the break period (*p* < 0.05). The mean levels of all work-related characteristics when excluding the break time were approximately 1 dBA higher than those when including the break periods (*p* < 0.001). However, the differences in mean levels when excluding or including the break periods for some job tasks, such as touch up, spray, power, and material classification, were relatively small but still statistically significant (*p* < 0.05).

**Figure 4:**
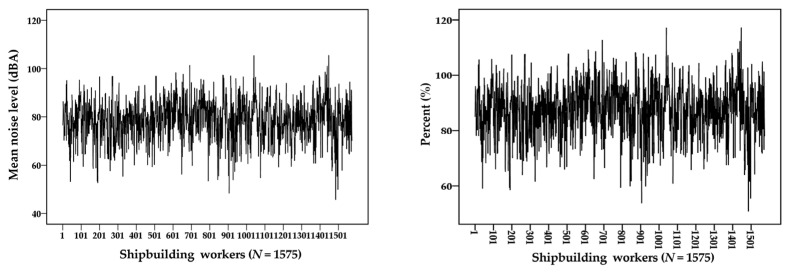
Mean noise levels and percent (%) for all subjects (*N* = 1575) during the shipbuilding processes.

Figure 4 also shows the mean noise levels and percentages (%) of the OEL (90 dBA) for all subjects (*N* = 1575) measured for three years (2016–2018). The mean noise exposure levels for all shipbuilding workers were approximately 88 dBA, and most samples did not exceed the OEL, but some peak exposure samples frequently exceeded the OEL. The noise exposure levels, converted to the percentage (%) compared to the OEL of 90 dBA, generally did not exceed a value of 100% but showed the pattern in which some measurements were exceeded.

Table 5 and Table 6 show the comparative results for the mean levels of occupational noise exposure when excluding and including the break periods using the KOSHA guide and U.S. OSHA method. In the case of excluding the break period, the overall mean level was 87.4 dBA using the KOSHA guide, which was significantly lower than the 88.3 dBA using OSHA’s method (*p* < 0.05) (Table 5). The number and proportion exceeding the level of OEL, 90 dBA, were also different between the KOSHA guide with 597 (37.9%) and the OSHA method with 629 (39.9%), respectively. Furthermore, the mean levels using these two methods were also significantly different by all characteristics (independent variables), including work environment, department, employment type, and job task only when excluding the break period.

In Table 6, when including the break time, the overall number and percentage exceeding the OELs, 90 dBA for KOSHA guide and 89.2 dBA for OSHA method, were significantly different between the KOSHA (*N* = 544, 34.5%) and OSHA method (*N* = 608, 38.6%) (*p* < 0.05). Among the work-related characteristics, the exceeding numbers and percentages were significantly different between the two guidelines for workplaces (outside and inside), department (subassembly), employment type (full-time and contractor workers), and occupation (welding and fit-up) in the McNemar test (*p* < 0.05).

Table 7 shows the mean levels of noise exposure collected for different daily monitoring schedules as it declined from more than 7 h (a reference value) to 4 h, and the intracorrelation coefficients were also calculated for each schedule compared to the reference value. According to the noise exposure assessment, the average noise level (*L_AVG_*) measured as a reference value was 88.3 dBA, which is between 0.75 and 1.0, the “very good” criterion of the ICC over all time periods with a measurement time of 4 to 6 h (unmeasured time of less than 3 h) [28]. The mean levels of noise exposure for several schedules of exposure monitoring collected for more than 6 h (<08:00–15:00, 08:00–15:00, 09:00–16:00) and for more than 5 h (08:00–14:00, 09:00–15:00) and 4 h per day (08:00–12:00, 09:00–14:00) were not significantly different from the reference value (*p* > 0.05). Most importantly, the mean levels with the number and proportion exceeding the exposure limit (90 dBA) during the two schedules, 09:00–16:00 (≥6 h) and 08:00–14:00 (≥5 h), were 88.3 dBA (*N* = 629, 39.9%) and 88.1 dBA (*N* = 628, 39.8%), respectively, almost equal to the reference value.

Table 8 also shows the mean levels of noise exposure collected for different daily monitoring schedules as the daily monitoring periods decreased from another reference value measured for more than 8 h (08:00–16:00), including the lunch break hour, and the number and proportions exceeding a modified exposure limit value of 89.2 dBA were compared. The ICC values of most daily schedules were between 0.75 and 1.00, suggesting “very good.” The mean levels of noise exposure for several monitoring schedules, <08:00–15:00 and 09:00–16:00 (≥7 h), and 08:00–14:00 and 09:00–15:00 (≥6 h), were not significantly different from the modified reference value (*p* > 0.05). The mean level (*L_AVG_*), 87.1 dBA (*N* = 610, 38.7%), collected during a schedule of 09:00–15:000 (≥6 h), was close to the modified reference value, 87.3 dBA.

## 4. Discussion

This study provides evidence that there is underestimation in the results using the KOSHA guideline, which suggests that the break periods (lunch break and preliminary preparation time to prepare for the noise exposure measurement) be taken out of the daily monitoring period when conducting noise exposure assessment in the shipbuilding processes in Korea. More importantly, there were significant differences in noise exposure levels measured in the shipbuilding workplaces depending on several work-related characteristics (e.g., measurement location, department, employment type, and detailed job tasks) when including or excluding lunch breaks and measurement preparation time. Therefore, noise exposure assessments, excluding the break periods during the exposure measurement time, could underestimate worker exposure levels because the proportions exceeding the OELs decreased. Furthermore, the exposure assessment, which increases the unmeasured time for occupational noise exposure by one hour, quantitatively demonstrated that it is best to measure continuously for at least 6 h (up to 3 p.m. or 4 p.m.) after starting the noise exposure measurement at 9 a.m., one hour after their job tasks.

Our study results showed three major findings. First, we conducted a noise exposure assessment according to the KOSHA guide and U.S. OSHA method during the break period (measurement preparation and lunchtime) and measured the cumulative noise exposure levels for a threshold level (TL) of 80 dBA. Of the total 1575 samples, high levels of noise exposure occurred out of 1432 (90.9%) preparation times for measurement and 1359 (86.9%) lunchtimes. These results indicate that the source of noise exposure exceeded the noise exposure limit during the break time during shipbuilding processes, and workers were indirectly exposed to noise generated during the break time when they were not wearing hearing protection equipment such as earplugs. Second, the noise exposure level decreased by approximately 1 dBA (lunchtime 0.8 dBA, preparation time 0.2 dBA) when the break time was included in the daily exposure measurement time, while the average noise level (*L_AVG_*) during the measurement time was significantly increased when the rest time was not included.

Furthermore, when comparing noise exposure levels, painting-related departments (prior and hull painting, etc.) and job tasks (spray, touch-up, power) were often exposed to relatively high levels of noise during the break periods, resulting in fewer differences from the average values than other job tasks. In fact, we observed many cases where the inspection was planned after the lunch break, and several job tasks, including blasting, power, spraying, and touch-up inside the painting shop blocks, engine rooms, and tanks, were inevitably carried out during the lunch break or after taking small lunch boxes in the actual workplaces. In the shipbuilding workplaces, a variety of hazardous agents (e.g., welding fume, metals, organic solvents, etc.), including noise, were mostly generated during several processes and operations, but the dilution ventilation system using air supply was employed. In this regard, high levels of background noise were generated since the air blower devices were installed near the site’s entrance and operated to quickly dry the painted ships and parts 24/7, which were the major source of increased noise exposure levels. Therefore, various job tasks related to the painting departments were shown to be more exposed to high levels of background noise, including noise from working on-site air tools and air emissions for painting, drying, or grinding on average, and were less different within the groups of workers. As shown in Table 5 and Table 6, the mean levels and exceeding number and percentages for most workers with some characteristics (e.g., work environment, department, and job tasks) were not significantly different between the KOSHA guide and OSHA method in the McNemar test.

Third, the noise exposure assessment strategies under the KOSHA guideline were underestimated by approximately 2% (excluding break time) and 4% (including break time) in the number and ratios exceeding the exposure limit compared to the U.S. OSHA method. The average noise level measured by the OSHA method was 88.3 dBA and 629 (39.9%), while the average noise level measured by the KOSHA guideline was 87.4 dBA and 597 (37.9%), respectively. When conducting the noise exposure assessment, including break times, the average noise level (*L_AVG_*) was the same, but the numbers and proportions exceeding the exposure limit value were underestimated by the KOSHA guide (34.5%) compared to the OSHA method (38.6%). These results were due to no modification of the occupational exposure limit for working hours exceeding 8 h in the KOSHA guide. Furthermore, the measurement time was reduced (i.e., an increase in the unmeasured time) to a baseline of exposure sampling with less than 1 hour of unmeasured time, and the results of the noise exposure monitoring time were the most reliable for 6 h (to 3 p.m. or 4 p.m.) after the beginning of the daily workday.

In a number of previous studies, daily working hours were found to be the most important determinant of occupational noise exposure along with each workload of job tasks, and a couple of studies quantitatively evaluating noise exposure levels during break hours have reported that there were significant effects on noise intensity, especially for shipbuilding processes, including high-frequency noise, indicating high levels of sound pressure in all workplace areas [29,30]. Severe, temporary hearing loss (TTS) results in longer recovery times, and the fastest recovery is within 30 or 60 min. Changes in hearing values over time after initial exposure have been reported to be faster than those without hearing protection [8,31]. Therefore, it is recommended to establish management and reduction measures for major noise sources during the break time. Another study suggested a method of calibrating abnormal tasks (working for more than 8 h) during a noise exposure assessment, using mathematical equations rather than calibrating occupational exposure criteria, to calibrate *DOSE* (%) during measurement time and then convert it to an average noise level for 8 h. In addition, the average and maximum 8-h TWA noise exposure values were 84.58 dBA and 93.01 dBA, respectively, and the percentage exceeding the Korean legal exposure limit was approximately 15.94% in the transport equipment manufacturing industry, including the shipbuilding industry. Decisions can be made based on the results of a noise exposure assessment to prevent noise exposure, such as hearing preservation programs at workplaces in the shipbuilding industry. For example, in the United States, all noise above 80 dBA should be measured to determine the need for hearing protection programs [32]. On the other hand, the Korean Ministry of Employment and Labor’s noise exposure limit is unclear regarding whether the exposure limits should be modified for noise work for at least 0.25 h (115 dBA) and up to 8 h (90 dBA). To prevent noise-induced heating loss, hearing protection devices (HPDs) should be continuously worn to reduce the exposure intensity and frequency from noise sources [33].

Our study has two strengths. First, noise exposure assessment was conducted for a total of 1575 full-time and contract workers employed at a large shipbuilding company in Korea. Face-to-face interviews were conducted with these workers during the noise exposure measurement in the morning of each day, and detailed work-related information about departments, workplaces, job tasks, and occupations was collected and reviewed. The collected qualitative information was also combined with the quantitative exposure data and recorded in a work environment monitoring report, which was used to classify individual workers into similar exposure groups (SEGs) and to evaluate and compare the average noise levels by each group. Furthermore, we characterized the exposure profile for each group of workers in the shipbuilding process under four different exposure scenarios: when excluding break times during the daily noise measurement time or when including break times, only preliminary preparation or lunchtimes. It is expected that exposure sources (e.g., grinder, tool noise, compressed air vent noise, alarm sound, etc.) can come from various job tasks during the break time. Using both qualitative and quantitative information to improve the quality of the working environment can reduce potential exposure to noise for other workers as much as possible.

Second, the average noise exposure level by job was quantitatively compared using two methods, the KOSHA guide in Korea and the OSHA method in the United States, and the average noise levels were calculated by abnormal work duration (over 8 h). In addition, the model simulations were conducted in two different situations, with or without break times, with an increase in the daily monitoring schedules by one hour. As a result, we were able to suggest the most relevant daily monitoring schedule for real-world workplaces in Korea. Using this approach, we derived similar results consistent with several previous studies, comprehensively identifying the work-related characteristics of all possible exposures during the daily working time and minimizing any systematic error or mistake in our noise exposure monitoring results.

However, our study has limitations. First, this is a case study based on a noise exposure assessment of some workers at large shipbuilding workplaces in a certain area in Korea, thus there are limits in generalizing our results to all manufacturing workers or theorizing about occupational noise exposure in other workplaces and different industries. Furthermore, we used historical exposure data recorded over the last three years from 2016 to 2018 and collected with the limited information on some determinants of occupational noise exposure for currently active workers in 2019, not former workers. Therefore, we have not fully investigated past work environment conditions, occupational exposure levels to various risk factors (e.g., dust, organic compounds, metals, etc.), other types of work performed during the processes, or other characteristics and patterns of noise exposure in the shipbuilding industry in the past. Nevertheless, the characteristics of break time during job tasks in the shipbuilding processes in Korea were quantitatively analyzed, and exposure profiles for occupational noise exposure classified by SEGs were also identified. Furthermore, it is of great significance that comprehensive noise exposure evaluation methods were presented by comparing the results using domestic and U.S. methods to identify the most appropriate daily exposure monitoring schedule (timing).

## 5. Conclusions

This study showed that workers employed at a large shipbuilding company in Korea were exposed to high levels of occupational noise during break periods, especially for those working in heating, grinding, and power processes in several painting-related departments. Furthermore, when conducting noise exposure assessments according to the KOSHA guide, we found evidence that exclusion of break times is insufficient; thus, occupational noise exposure levels were underestimated. By comparison with the results of the exposure assessment using the U.S. OSHA’s method, we suggest that the most reliable schedule of daily noise exposure measurement should include the break time and must be measured continuously for at least six consecutive hours after the beginning of the job tasks. Therefore, it is necessary to conduct further studies to identify the most integrated exposure assessment strategy and daily monitoring schedule that are applicable to other workers with various job tasks in different industries in the future.

## Figures and Tables

**Table 1:** Characteristics of work environment, department, employment type, and occupation (job task) in the shipbuilding process in Korea.

Characteristics	Description
Work environment	
Outside	Job tasks performed outside the workplace (e.g., hull assembly, outfitting, hull painting, sea trial)
Inside	Job tasks performed inside the workplace (e.g., subassembly, panel assembly, unit assembly, pre-outfitting, pre-painting)
Combined	Job tasks performed both outside and inside the workplace (e.g., sub-departments)
Department	
Hull assembly	The completed larger units are then moved to the graving dock, shipway or final assembly area. Here, the larger units are joined together to form the vessel
Hull painting	Hull surface preparation and painting on repair ships is normally performed when the ship is fully drydocked (i.e., on the graving dock of a floating drydock).
Sea trial	After completion of the outfitting phase, the ship undergoes both dock and sea trials, during which all the ship’s systems are proved to be fully functional and operational.
Outfitting	The process of installing parts and various subassemblies (e.g., piping systems, ventilation equipment, electrical components) on the block prior to joining the blocks together at erections. After the ship is launched, it enters the outfitting phase. A large amount of time and equipment are required. The work includes the fitting of cabling and piping, the furnishing of galleys and accommodations, insulation work, installation of electronic equipment and navigation aids, and installation of propulsion and ancillary machinery.
Subassembly	The basic component of shipbuilding is steel plate. The plates are cut, shaped, bent or otherwise manufactured to the desired configuration specified by the design.
Panel assembly	The plates are joined into various units and subassemblies. At this juncture, piping, electrical, and other utility systems are assembled and integrated into the units. The units are assembled using automatic or manual welding or a combination of the two.
Unit assembly	The units or subassemblies are usually then transferred to an open-air platen or lay down area where erection, or joining of assemblies, occurs to form even larger units or blocks
Block painting	blasted to ensure proper profiling, and painted
Misc.	The rest of the departments
Employment type	
Full-time worker	Directly employed by the shipbuilding company
Contractor worker	Affiliated to several vendor companies
Occupation (job task)	
Blasting	Removing and cleaning substances, including scale, rust, etc., on the surface of the block
Power	Removing paint, rust, etc. applied to parts requiring modification outside of a ship
Spray	Spraying paint using a compressed air gun
Touch up	Touching up paint using a small brush and pulley
Heating	Bending an iron plate by heating
Grinder	Cutting and grinding scales of weld metal parts
Sea trial	Sea trial commander responsible for managing the vessel and all personnel on board
Signal	Signaling for crane, forklift truck, etc.
Welding	Joining and welding between the steel plates by heating and melting
Material classification	Preparation and classification of raw materials
Cutting	Cutting steel plates into desired shapes and sizes
Fit-up	Fitting up steel plates prior to pre-welding
Misc.	The rest of job tasks

**Table 2:** Comparison of noise exposure assessment methods between the U.S. OSHA method and KOSHA guide.

Methodology	Including Entire Break Time ^1^ (9 h)			Excluding Entire Break Time ^2^ (8 h)		
	Time (1 h)	*DOSE* (%)	OEL	Time (1 h)	*DOSE* (%)	OEL
OSHA method	Included	Included	89.2 dBA	Excluded	Excluded	90 dBA
KOSHA guide	Included	Included	90 dBA	Excluded	Included	90 dBA

^1^ *L_AVG_* is calculated including two values of Time (1 h) and *DOSE* (%) in Equation (2) when using the U.S. OSHA method and KOSHA guide. ^2^ *L_AVG_* is calculated excluding the two values of Time (1 h) and *DOSE* (%) when using the U.S. OSHA method, but *DOSE* (%) was only included in Equation (2) when using the KOSHA guide.

**Table 3:** The measured results of occupational noise exposure during the break periods in the shipbuilding workplaces.

Characteristics	Preparation Time					Lunch Break				
	*N* ^1^	Time (h) ^2^	*DOSE* (%) ^3^	Max	*L_AVG_* ^4^	*N* ^1^	Time (h) ^2^	*DOSE* (%) ^3^	Max	*L_AVG_* ^4^
Overall	1432	0.4	0.9	110.0	77.6	1359	1	6.1	125.9	84.8
Work environment										
Outside	583	0.4	0.4	91.4	71.8	548	1	5.1	125.9	83.5
Inside	635	0.3	1.2	110.0	81.8	617	1	2.2	114.2	77.5
Outside/Inside	214	0.4	0.7	96.2	75.8	194	1	0.9	89.5	71.0
Department										
Hull assembly	250	0.4	0.5	91.4	73.4	233	1	1.8	106.0	76.0
Hull painting	72	0.4	0.4	80.8	71.8	67	1	28.5	125.9	95.9
Sea trial	47	0.4	0.3	75.6	69.7	48	1	0.6	81.8	68.1
Outfitting	214	0.4	0.4	90.9	71.8	200	1	2.3	106.3	77.8
Subassembly	173	0.2	0.7	101.5	80.8	175	1	1.7	100.1	75.6
Panel assembly	186	0.3	0.9	101.1	79.7	173	1	1.0	88.4	71.8
Unit assembly	195	0.4	0.5	100.6	73.4	188	1	0.9	91.1	71.0
Block painting	81	0.4	4.3	110.0	88.9	81	1	9.0	114.2	87.6
Misc.	214	0.4	0.7	96.2	75.8	194	1	0.9	89.5	71.0
Employment type										
Full-time worker	591	0.3	0.4	100.6	73.9	542	1	0.8	87.4	70.2
Contractor worker	841	0.4	1.1	110.0	79.1	817	1	4.8	125.9	83.1
Occupation (job task)										
Blasting	18	0.5	3.1	96.0	84.9	16	1	5.8	98.3	84.5
Power	41	0.4	0.6	85.0	74.7	37	1	56.4	125.9	100.9
Spray	57	0.4	0.4	81.8	71.8	56	1	1.0	87.1	71.8
Touch up	24	0.5	1.1	87.2	77.5	22	1	1.6	84.8	75.2
Heating	28	0.3	0.6	86.5	76.8	28	1	1.6	91.1	75.2
Grinder	159	0.4	2.4	110.0	84.7	154	1	6.0	114.2	84.7
Sea trial	23	0.4	0.4	75.6	71.8	24	1	0.8	81.8	70.2
Signal	27	0.4	0.6	85.3	74.7	25	1	0.6	77.9	68.1
Welding	318	0.3	0.5	100.6	75.5	281	1	1.1	86.8	72.5
Material classification	93	0.3	0.6	89.5	76.8	85	1	0.9	81.4	71.0
Cutting	43	0.2	0.3	83.9	74.7	44	1	1.2	85.5	73.1
Fit-up	390	0.4	0.6	101.5	74.7	376	1	1.2	100.1	73.1
Misc.	211	0.4	0.5	96.2	73.4	211	1	0.8	89.5	70.2

^1^ Subjects, who had no value of *DOSE* (%) (*N* = 143 for preparation, *N* = 216 for lunch break), were excluded in the analysis. ^2^ Average of hour, *DOSE* (%). ^3^ Average of *DOSE* (%). ^4^ Average obtained from time and *DOSE* (%), dBA.

**Table 4:** Comparison of the mean levels of occupational noise exposure depending on inclusion or exclusion of the entire break periods.

Characteristics	*N*	Mean Levels of Occupational Noise Exposure [Average of *L_AVG_* (Standard Deviation), Unit: dBA]		*p*-Value ^1^
		Excluding Entire Break Time (8 h)	Including Entire Break Time (9 h)	
Overall	1575	88.3 (8.0)	87.3 (7.9)	<0.001
Work environment				
Outside	658	87.5 (7.7)	86.5 (7.6)	<0.001
Inside	679	90.6 (7.6)	89.6 (7.5)	<0.001
Outside/Inside	238	84.0 (7.8)	83.0 (7.6)	<0.001
Department				
Hull assembly	291	89.7 (7.6)	88.6 (7.5)	<0.001
Hull painting	77	87.5 (8.0)	86.6 (8.0)	<0.001
Sea trial	54	81.4 (6.1)	80.4 (6.0)	<0.001
Outfitting	236	86.1 (7.0)	85.1 (7.0)	<0.001
Subassembly	188	90.7 (7.9)	89.7 (7.8)	<0.001
Panel assembly	191	90.0 (7.1)	88.9 (7.0)	<0.001
Unit assembly	213	92.0 (6.7)	90.9 (6.6)	<0.001
Block painting	87	88.6 (8.9)	87.8 (9.0)	<0.001
Misc.	238	84.0 (7.8)	83.0 (7.6)	<0.001
Employment type				
Full-time worker	667	86.3 (7.4)	85.3 (7.3)	<0.001
Contractor worker	908	89.8 (8.1)	88.8 (8.0)	<0.001
Occupation (job task)				
Blasting	18	90.6 (7.5)	89.6 (7.5)	<0.001
Power	45	93.7 (7.4)	92.8 (7.7)	<0.001
Spray	61	82.7 (6.1)	81.8 (6.1)	<0.001
Touch up	25	81.8 (3.7)	81.1 (3.7)	<0.001
Heating	30	100.8 (5.4)	99.6 (5.3)	<0.001
Grinder	172	97.3 (6.5)	96.2 (6.4)	<0.001
Sea trial	24	80.7 (6.8)	79.8 (6.6)	<0.001
Signal	30	81.9 (4.6)	80.9 (4.5)	<0.001
Welding	349	89.1 (5.2)	88.1 (5.2)	<0.001
Material classification	100	80.6 (6.4)	79.9 (6.2)	<0.001
Cutting	46	90.6 (3.8)	89.6 (3.8)	<0.001
Fit-up	434	89.3 (7.1)	88.2 (7.1)	<0.001
Misc.	241	82.6 (6.0)	81.4 (1.1)	<0.001

^1^ Paired samples *t*-test for continuous variable data between groups (*p* < 0.05).

**Table 5:** Comparison of the mean noise exposure levels by KOSHA and OSHA method (when excluding break time, 8 h).

Characteristics	*N*	KOSHA Guide		OSHA Method		*p*-Value ^2^	*p*-Value ^3^
		Mean (SD) ^1^	>90 dBA [*N* (%)]	Mean (SD)^1^	>90 dBA [*N* (%)]		
Overall	1575	87.4 (8.5)	597 (37.9)	88.3 (8.0)	629 (39.9)	<0.001	<0.001
Work environment							
Outside	658	86.5 (8.3)	220 (33.4)	87.5 (7.7)	231 (35.1)	<0.001	0.005
Inside	679	89.9 (7.9)	336 (49.5)	90.6 (7.6)	354 (52.1)	<0.001	<0.001
Outside/Inside	238	82.8 (8.2)	41 (17.2)	84.0 (7.8)	44 (18.5)	<0.001	0.083
Department							
Hull assembly	291	88.9 (8.1)	128 (44.0)	89.7 (7.6)	133 (45.7)	<0.001	0.059
Hull painting	77	86.6 (8.6)	24 (31.2)	87.5 (8.0)	26 (33.8)	<0.001	0.157
Sea trial	54	80.0 (6.7)	3 (5.6)	81.4 (6.1)	3 (5.6)	<0.001	1.000
Outfitting	236	85.1 (7.5)	65 (27.5)	86.1 (7.0)	69 (29.2)	<0.001	0.102
Subassembly	188	90.1 (8.3)	103 (54.8)	90.7 (7.9)	106 (56.4)	<0.001	0.083
Panel assembly	191	89.1 (7.7)	82 (42.9)	90.0 (7.1)	92 (48.2)	<0.001	0.002
Unit assembly	213	91.3 (6.8)	118 (55.4)	92.0 (6.7)	123 (57.7)	<0.001	0.025
Block painting	87	87.8 (9.4)	33 (37.9)	88.6 (8.9)	33 (37.9)	<0.001	1.000
Misc.	238	82.8 (8.2)	41 (17.2)	84.0 (7.8)	44 (18.5)	<0.001	0.083
Employment type							
Full-time worker	667	85.3 (8.1)	187 (28.0)	86.3 (7.4)	204 (30.6)	<0.001	<0.001
Contractor worker	908	89.0 (8.4)	410 (45.2)	89.8 (8.1)	425 (46.8)	<0.001	0.001
Occupation (job task)							
Blasting	18	89.8 (7.7)	8 (44.4)	90.6 (7.5)	8 (44.4)	<0.001	1.000
Power	45	93.2 (8.0)	33 (73.3)	93.7 (7.4)	33 (73.3)	<0.001	1.000
Spray	61	81.5 (6.7)	6 (9.8)	82.7 (6.1)	6 (9.8)	<0.001	1.000
Touch up	25	80.7 (4.0)	1 (4.0)	81.8 (3.7)	1 (4.0)	<0.001	1.000
Heating	30	100.3 (5.4)	29 (96.7)	100.8 (5.4)	29 (96.7)	<0.001	1.000
Grinder	172	96.7 (6.6)	147 (84.9)	97.3 (6.5)	149 (86.6)	<0.001	0.180
Sea trial	24	79.1 (7.5)	1 (4.2)	80.7 (6.8)	1 (4.2)	<0.001	1.000
Signal	30	80.1 (6.0)	1 (3.3)	81.9 (4.6)	2 (6.7)	<0.001	0.317
Welding	349	88.5 (5.5)	124 (35.5)	89.1 (5.2)	137 (39.3)	<0.001	<0.001
Material classification	100	78.9 (7.4)	7 (7.0)	80.6 (6.4)	7 (7.0)	<0.001	1.000
Cutting	46	90.2 (3.8)	26 (56.5)	90.6 (3.8)	27 (58.7)	<0.001	0.317
Fit-up	434	88.5 (7.4)	190 (43.8)	89.3 (7.1)	200 (46.1)	<0.001	0.004
Misc.	241	81.5 (6.4)	25 (10.4)	82.6 (6.0)	29 (12.0)	<0.001	0.046

^1^ Average of *L_AVG_* (standard deviation), dBA. ^2^ Paired sample t-test for continuous data between groups (*p* < 0.05). ^3^ McNemar test for categorical data within groups (*p* < 0.05).

**Table 6:** Comparison of the mean noise exposure levels by KOSHA and OSHA method (when including break time, 9 h).

Characteristics	*N*	Mean (SD) ^1^	KOSHA Guide	OSHA Method	*p*-Value ^2^
			>90 dBA [*N* (%)]	>89.2 dBA [*N* (%)]	
Overall	1575	87.3 (7.9)	544 (34.5)	608 (38.6)	<0.001
Work environment					
Outside	658	86.5 (7.6)	203 (30.9)	223 (33.9)	<0.001
Inside	679	89.6 (7.5)	302 (44.5)	342 (50.4)	<0.001
Outside/Inside	238	83.0 (7.6)	39 (16.4)	43 (18.1)	0.046
Department					
Hull assembly	291	88.6 (7.5)	117 (40.2)	129 (44.3)	0.001
Hull painting	77	86.6 (8.0)	23 (29.9)	24 (31.2)	0.317
Sea trial	54	80.4 (6.0)	3 (5.6)	3 (5.6)	1.000
Outfitting	236	85.1 (7.0)	60 (25.4)	67 (28.4)	0.008
Subassembly	188	89.7 (7.8)	86 (45.7)	104 (55.3)	<0.001
Panel assembly	191	88.9 (7.0)	76 (39.8)	85 (44.5)	0.003
Unit assembly	213	90.9 (6.6)	109 (51.2)	120 (56.3)	0.001
Block painting	87	87.8 (9.0)	31 (35.6)	33 (37.9)	0.157
Misc.	238	83.0 (7.6)	39 (16.4)	43 (18.1)	0.046
Employment type					
Full-time worker	667	85.3 (7.3)	165 (24.7)	193 (28.9)	<0.001
Contractor worker	908	88.8 (8.0)	379 (41.7)	415 (45.7)	<0.001
Occupation (job task)					
Blasting	18	89.6 (7.5)	7 (38.9)	8 (44.4)	0.317
Power	45	92.8 (7.7)	32 (71.1)	33 (73.3)	0.317
Spray	61	81.8 (6.1)	6 (9.8)	6 (9.8)	1.000
Touch up	25	81.1 (3.7)	1 (4.0)	1 (4.0)	1.000
Heating	30	99.6 (5.3)	29 (96.7)	29 (96.7)	1.000
Grinder	172	96.2 (6.4)	143 (83.1)	147 (85.5)	0.046
Sea trial	24	79.8 (6.6)	1 (4.2)	1 (4.2)	1.000
Signal	30	80.9 (4.5)	1 (3.3)	1 (3.3)	1.000
Welding	349	88.1 (5.2)	100 (28.7)	127 (36.4)	<0.001
Material classification	100	79.9 (6.2)	5 (5.0)	7 (7.0)	0.157
Cutting	46	89.6 (3.8)	17 (37.0)	26 (56.5)	0.003
Fit-up	434	88.2 (7.1)	179 (41.2)	195 (44.9)	<0.001
Misc.	241	81.4 (1.1)	24 (10.0)	27 (11.2)	0.083

^1^ Average of *L_AVG_* (standard deviation), dBA. ^2^ McNemar test for categorical data within groups (*p* < 0.05).

**Table 7:** The mean levels of occupational noise exposure compared with an exposure limit of 90 dBA (8 h), excluding break period, by increasing an hour in different monitoring schedules.

*L_AVG_* (N = 1575)	Mean (SD) ^1^	ICC ^2^	>90 dBA (8 h)		*p*-Value ^3^
			N	%	
Reference value	88.3 (8.0)	-	629	39.9	-
More than 6 h					
<08:00~15:00	88.0 (8.1)	0.994	616	39.1	0.085
08:00~15:00	88.3 (8.1)	0.994	644	40.8	0.063
09:00~16:00	88.3 (8.2)	0.995	629	39.9	1.000
More than 5 h					
<08:00~14:00	87.7 (8.2)	0.984	594	37.7	0.001
08:00~14:00	88.1 (8.3)	0.984	628	39.8	0.922
09:00~15:00	88.2 (8.3)	0.989	635	40.3	0.508
10:00~16:00	87.9 (8.4)	0.984	597	37.9	0.001
More than 4 h					
<08:00~12:00	87.6 (8.3)	0.967	574	36.4	<0.001
08:00~12:00	88.0 (8.4)	0.967	616	39.1	0.256
09:00~14:00	88.0 (8.5)	0.978	614	38.9	0.166
10:00~15:00	87.8 (8.6)	0.978	607	38.5	0.036
11:00~16:00	87.4 (8.7)	0.958	581	36.8	0.000

^1^ Average of *L_AVG_* (standard deviation), dBA. ^2^ ICC (Intraclass correlation coefficients) for continuous variable data within groups. ^3^ McNemar test for categorical data within groups (*p* < 0.05).

**Table 8:** The mean levels of occupational noise exposure compared with an exposure limit of 89.2 dBA (9 h), including break period, by increasing an hour in different monitoring schedules.

*L_AVG_* (N = 1575)	Mean (SD) ^1^	ICC ^2^	>89.2 dBA (9 h)		*p*-Value ^3^
			N	%	
Reference value	87.3 (7.9)	-	608	38.6	-
More than 7 h					
<08:00~15:00	87.1 (8.0)	0.994	598	38.0	0.140
08:00~15:00	87.4 (8.1)	0.994	627	39.8	0.018
09:00~16:00	87.3 (8.1)	0.994	612	38.9	0.579
More than 6 h					
<08:00~14:00	86.7 (8.1)	0.985	567	36.0	<0.001
08:00~14:00	87.0 (8.2)	0.984	593	37.7	0.128
09:00~15:00	87.1 (8.3)	0.989	610	38.7	0.816
10:00~16:00	86.8 (8.3)	0.984	570	36.2	<0.001
More than 5 h					
09:00~14:00	86.6 (8.4)	0.978	565	35.9	<0.001
10:00~15:00	86.5 (8.5)	0.978	553	35.1	<0.001
11:00~16:00	86.1 (8.6)	0.958	522	33.1	<0.001

^1^ Average of *L_AVG_* (standard deviation), dBA. ^2^ ICC (Intraclass correlation coefficients) for continuous variable data within groups. ^3^ McNemar test for categorical data within groups (*p* < 0.05).

## Data Availability

The data presented in this study are available from the corresponding author upon reasonable request.

## References

[1] Nelson D.I., Nelson R.Y., Concha-Barrientos M., Fingerhut M. (2005). The global burden of occupational noise-induced hearing loss. Am. J. Ind. Med..

[2] Lee J.H. (2010). Occupational diseases of noise exposed workers. Hanyang Med. Rev..

[3] Basner M., Babisch W., Davis A., Brink M., Clark C., Janssen S., Stansfeld S. (2014). Auditory and non-auditory effects of noise on health. Lancet.

[4] Jang E.C. (2018). Occupational Noise Exposure and Serum Lipids in Manufacturing Company Workers. Soonchunhyang Med. Sci..

[5] Oh M., Shin K., Kim K., Shin J. (2019). Influence of noise exposure on cardiocerebrovascular disease in Korea. Sci. Total Environ..

[6] Khosravipour M., Abdollahzad H., Khosravi F., Rezaei M., Mohammadi Sarableh H., Moradi Z. (2020). The Association of Occupational Noises and the Prevalence of Metabolic Syndrome. Ann. Work Expo. Health.

[7] Kim M.G., Ahn Y.-S. (2021). The relationship between occupational noise exposure and hypertension using nearest age-matching method in South Korea male workers. Cogent Eng..

[8] Kang B., Kim H. (1983). Recovery From Tempory Threshold Shifts after Short Term Exposure to Various Noise. Ocean Underw. Med..

[9] Kwak M., Lee J., Kim J., Urm S., Kim D., Son B., Lee C. (1997). Evaluation on Hearing Conservation Program in the Noisy Industries. J. Prev. Med. Public Health.

[10] Jang J.-K., Chung K.-J. (2008). Work environment management and measurement measures for reducing occupational noise exposure. Occup. Health.

[11] Tao L., Zeng L., Wu K., Zhang H., Wu J., Zhao Y., Li N., Zhao Y. (2016). Comparison of four task-based measurement indices with full-shift dosimetry in a complicated noise environment. Int. J. Ind. Ergon..

[12] KOSHA (2016). Occupational noise exposure assessment in the workplaces. KOSHA Guide.

[13] Driscoll D.P. (2005). Noise exposure assessment for extended work shifts: What are the options. UPDATE.

[14] Kim K.-Y., Kang T., Lee S.G., Park H.D., Jeong J.Y. (2017). A review of a system for improving the reliability of domestic measurement results regarding the work environment. J. Korean Soc. Occup. Environ. Hyg..

[15] Jeong J.Y., Kang T.S., Lee S.G., Park H.D., Kim K.Y. (2017). An improvement plan for a workplace monitoring system through random selection of workplaces and unnoticed measurement inspection. J. Korean Soc. Occup. Environ. Hyg..

[16] Sayler S.K., Roberts B.J., Manning M.A., Sun K., Neitzel R.L. (2019). Patterns and trends in OSHA occupational noise exposure measurements from 1979 to 2013. Occup. Environ. Med..

[17] Hwang G. (2020). A Study on the Registration of Workplaces subjected to Work Environment Measurement to Expand it’s Coverage. J. Korean Soc. Occup. Environ. Hyg..

[18] Lawton B.W. (2003). Commentary: The 1971 BOHS Hygiene Standard for Wide-band Noise. Ann. Occup. Hyg..

[19] AIHA (1994). American Industrial Hygiene Association White Paper: A Generic Exposure Assessment Standard. Am. Ind. Hyg. Assoc. J..

[20] OSHA, U.S (2013). OSHA Technical Manual (OTM) Section III: Chapter 5—Noise.

[21] Seixas N.S., Sheppard L., Neitzel R. (2003). Comparison of task-based estimates with full-shift measurements of noise exposure. AIHA J..

[22] Brueck S.E., Prince Panaccio M., Stancescu D., Woskie S., Estill C., Waters M. (2013). Noise Exposure Reconstruction and Evaluation of Exposure Trends in Two Large Automotive Plants. Ann. Occup. Hyg..

[23] Middendorf P.J. (2004). Surveillance of occupational noise exposures using OSHA’s Integrated Management Information System. Am. J. Ind. Med..

[24] Roh Y., Yim H., Kim S., Park H., Jung J., Park S., Kim H., Chung C., Lee W. (2001). Recommendation and current status in exposure assessment using monitoring data in ship building industry—Focused on the similar exposure group(SEG). J. Korean Soc. Occup. Environ. Hyg..

[25] Song J.-H., Hong S.-Y., Lee Y.-S., Kwon H.-W. (2015). Computational Analysis on the Noise Characteristics of Ship Large Duct. J. Korean Soc. Mar. Environ. Saf..

[26] Huh J.-H., Cho K.-S., Ahn K.-D., Lee B.-K. (2000). Estimation of Noise Exposure dureation by a Noise Dosimeter. Soonchunhyang Med. Sci. (SMS).

[27] Choe H.-G., Shin Y.-I., Yang B.-S., Lee Y.-W., Kim K.-H. (2003). A Study on the Characteristics of Noise in Small Boats. J. Korea Ship Safrty Technol. Auth..

[28] Kong K.A. (2017). Statistical methods: Reliability assessment and method comparison. Ewha Med. J..

[29] Bhumika N., Prabhu G., Ferreira A., Kulkarni M. (2013). Noise-Induced Hearing Loss Still a Problem in Shipbuilders: A Cross. Sectional Study in Goa, India. Ann. Med. Health Sci. Res..

[30] Alexopoulos E.C., Tsouvaltzidou T. (2015). Hearing loss in shipyard employees. Indian J. Occup. Environ. Med..

[31] Kim J.-M. (2007). Effects of occupational noise exposure on human health in the work environments. Korean Soc. Noise Vib. Eng..

[32] Tak S., Davis R.R., Calvert G.M. (2009). Exposure to hazardous workplace noise and use of hearing protection devices among US workers—NHANES, 1999–2004. Am. J. Ind. Med..

[33] Song H., Jeong S., Lee E., Alsabbagh N., Lee J., You S., Kwak C., Kim S., Han W. (2019). Types of Hearing Protection Devices and Application. Korean J. Otorhinolaryngol. Head Neck Surg..

